# Antibacterial Activity and Cell Viability of Biomimetic Magnesian Calcite Coatings on Biodegradable Mg

**DOI:** 10.3390/jfb14020098

**Published:** 2023-02-10

**Authors:** Monica Popa, Mihai Anastasescu, Laura M. Stefan, Ana-Maria Prelipcean, Jose Calderon Moreno

**Affiliations:** 1 Oxide Compounds and Materials Science Laboratory, “Ilie Murgulescu” Institute of Physical Chemistry, 202 Splaiul Independentei, 060021 Bucharest, Romania; 2 Surface Chemistry and Catalysis Laboratory, “Ilie Murgulescu” Institute of Physical Chemistry, 202 Splaiul Independentei, 060021 Bucharest, Romania; 3 Department of Cellular and Molecular Biology, National Institute of R&D for Biological Sciences, 296 Splaiul Independentei, 060031 Bucharest, Romania

**Keywords:** resorbable biomaterial, CaCO_3_, amorphous calcium carbonate (ACC), corrosion protective film, antibacterial, cell viability, bone scaffolds

## Abstract

Mg is a material of choice for biodegradable implants. The main challenge for using Mg in temporary implants is to provide protective surfaces that mitigate its rapid degradation in biological fluids and also confer sufficient cytocompatibility and bacterial resistance to Mg-coated surfaces. Even though carbonate mineralization is the most important source of biominerals, such as the skeletons and shells of many marine organisms, there has been little success in the controlled growth of carbonate layers by synthetic processes. We present here the formation mechanism, antibacterial activity, and cell viability of magnesian calcite biomimetic coatings grown on biodegradable Mg via a green, one-step route. Cell compatibility assessment showed cell viability higher than 80% after 72 h using fibroblast cells (NCTC, clone L929) and higher than 60% after 72 h using human osteoblast-like cells (SaOS-2); the cells displayed a normal appearance and a density similar to the control sample. Antimicrobial potential evaluation against both Gram-positive (*Staphylococcus aureus* (ATCC 25923)) and Gram-negative (*Pseudomonas aeruginosa* (ATCC 27853)) strains demonstrated that the coated samples significantly inhibited bacterial adhesion and biofilm formation compared to the untreated control. Calcite coatings grown on biodegradable Mg by a single coating process showed the necessary properties of cell compatibility and bacterial resistance for application in surface-modified Mg biomaterials for temporary implants.

## 1. Introduction

Mg has excellent biomechanical compatibility with human bone, but its use as a fixation device and scaffold for tissue engineering is hampered by a rapid degradation rate in the physiological environment [1,2,3]. Hybrid layer coatings, with a first porous oxide layer obtained by plasma electrolytic oxidation and a second layer subsequently deposited by sol-gel or polymer coating, have previously demonstrated to be a good method for decreasing the rate of corrosion [4,5,6,7]. Oxide-carbonate coatings have also demonstrated self-healing performance on the surface of AZ41 Mg alloy via ultrasound-assisted chemical conversion [8]. Several studies [9,10,11,12,13,14,15,16] have reported the development of single-layer carbonate coatings to control the corrosion of Mg and alloys: a protective MgCO_3_ layer was obtained by electron beam irradiation inside an environmental TEM [9], a continuous protective film of nesquehonite was grown directly on Mg in humid CO_2_ at 40 °C and 65 atm in a pressure autoclave [10], and protective layers of Mg_5_(CO_3_)_4_(OH)_2_·4H_2_O [11] and CaCO_3_ [12] were obtained by hydrothermal synthesis. Recently, Ca(Mg)CO_3_ coatings providing a much improved corrosion resistance in isotonic physiological fluids containing chloride ions were grown on pure Mg [13] and on AZ91 Mg alloy [14] via simple green conversion methods in aqueous solution; on Mg2Zn0.2Ca alloy via hydrothermal method [12,15]; and on Mg-Nd alloy by ultrasound-assisted chemical conversion [17]. CaCO_3_ does not pose a threat to any species and is therefore greatly biocompatible [18] and widely used as a biomaterial in many products, such as toothpaste [19] and cosmetics [20], as a vector to deliver drugs, genes, and enzymes [21], and for controlled degradability in vivo [22]. Therefore, the availability of simple, green, synthetic methods for protective carbonate coatings on Mg and alloys opens up the possibility of their application in Mg-based resorbable implants, which could allow the use of synthetic scaffolds for regeneration and restoration of bone function [23] or of resorbable fixation screws without the need for a second surgery for extraction [24], which are current topics in biomaterials research. 

The present study aims to gain insight into the formation and growth of calcium carbonate films by the one-step, single-pot method of immersion in carbonated water and also to evaluate the cytocompatibility and antibacterial properties of the calcium carbonate films grown on pure Mg. We used Raman vibrational spectroscopy, SEM, and AFM to determine the phase and morphological evolution with time of Mg immersed in carbonated water to modify the surface with a calcite coating. A tentative mechanism for CaCO_3_ coating formation and growth on Mg surfaces in carbonate is proposed. In addition, cell viability assessment using fibroblast cells (NCTC, clone L929) and human osteoblast-like cells (SaOS-2) were carried out by MTT assay and lactate dehydrogenase (LDH) release; cell morphology was analyzed by optical and fluorescence microscopy to determine cytotoxicity. The antimicrobial potential of carbonate coatings against both Gram-positive (*Staphylococcus aureus* (ATCC 25923)) and Gram-negative (*Pseudomonas aeruginosa* (ATCC 27853)) strains was evaluated for the antibacterial effect on planktonic growth and antibiofilm activity. The calcium carbonate (CaCO_3_) films grown by aqueous solution chemistry on magnesium using a simple, single-step, low-cost process demonstrated good cell compatibility and antibacterial behavior and have high potential to be used as a non-toxic corrosion barrier on Mg-based resorbable implants.

## 2. Materials and Methods

### 2.1. Material 

Magnesium rods of high purity (99.94 wt.% Mg) were used. Disks of 15 mm diameter and 2 mm thickness were cut, cleaned with ethanol, and dried at room temperature before coating. Optical images were obtained with a digital camera with optical zoom.

### 2.2. Coating

The pure Mg samples were immersed in the coating solution, carbonated water from Romaqua Group (7.7 × 10^−3^ mol/L Ca^2+^; 31.0 × 10^−3^ mol/L HCO_3_^−^; 4.7 × 10^−3^ mol/L Mg^2+^; 3.6 × 10^−3^ mol/L Na^+^; and 56.8 × 10^−3^ mol/L CO_2_), at room temperature. The evolution of carbonate coating was evaluated for immersion times of 5, 10, 15, 20, 25, 30, 40, 50, and 60 min. The samples were allowed to air dry after immersion and were further analyzed.

### 2.3. Structure Characterization 

Raman spectra were recorded in a Horiba Jobin Yvon LabRam HR spectrometer using a 325 nm excitation laser. The spectra were recorded in the region 400–4000 cm^−1^ by adding three successive measurements using an integration time of 30 s per measurement and a total acquisition time of 90 s for each full spectrum. A high-resolution microscope Quanta3D FEG operating between 2 and 10 kV was used for the morphological studies by scanning electron microscopy (SEM). Atomic force microscopy (AFM) measurements were recorded using an XE100 microscope (Park Systems, Suwon, Republic of Korea) equipped with flexure-guided, cross-talk-eliminated scanners. All images were recorded with sharp PPP-NCHR tips (Nanosensors^TM^) with less than 10 nm tip radius of curvature (typically 8 nm), approx. 125 μm length, approx. 30 μm width, approx. 42 N/m force constant, and approx. 330 kHz resonance frequency. “True non-contact” working mode was used to minimize tip–sample interaction. AFM images were processed with the XEI program (v 1.8.0—Park Systems) for roughness evaluation and the SPIP program (Version 4.6.0.0) developed by Image Metrology, and are presented in “enhanced color”™ viewing mode to highlight morphological details. Representative line scans were selected, showing the surface profiles in detail. The root-mean-squared roughness (Rq) represents the standard deviation of the height value in the image, while the peak-to-valley parameter (Rpv) is the height difference between the lowest and highest point.

### 2.4. Cell Culture Experiments: In Vitro Cytotoxicity Tests 

Murine fibroblast cells (NCTC, clone L929) and human osteoblast-like cells (SaOS-2) were used to evaluate in vitro potential cytotoxic effects. NCTC cells were grown in Minimum Essential Medium (MEM) supplemented with 10% fetal bovine serum (FBS) and 1% antibiotics (penicillin, streptomycin, and neomycin), whereas SaOS-2 cells were grown in DMEM:Ham’s F12 (1:1 mixture) supplemented with 10% FBS and 1% antibiotics. Both cell lines were maintained at 37 °C in a humidified atmosphere with 5% CO_2_. The cytotoxicity tests were performed by indirect contact assay according to the ISO 10993-5 standard. Sample extracts were prepared in a serum-free culture medium with a surface/volume ratio of 1 cm^2^/mL in a humidified atmosphere with 5% CO_2_ at 37 °C for 72 h. Cells were seeded in 100 µL culture medium at a density of 5 × 10^4^ cells/mL in 96-well cell culture plates and incubated for 24 h to allow cell attachment. The culture medium was then replaced with the extracts at different dilutions: 1-fold (100 µL extract; undiluted extract), 2-fold (50 µL extract and 50 µL culture medium), 5-fold (20 µL extract and 80 µL culture medium), and 10-fold (10 µL extract and 90 µL culture medium). After cell incubation in standard conditions for 24 h and 72 h, quantitative 3-(4,5-dimethylthiazol-2-yl)-2,5-diphenyltetrazolium bromide (MTT) and lactate dehydrogenase (LDH) assays were performed. Cell morphology was also evaluated by fluorescence microscopy after staining with specific dyes.

To evaluate the cell viability, treated cells were incubated with 0.25 mg/mL MTT working solution (Sigma-Aldrich, Saint Louis, MO, USA) for 3 h at 37 °C, according to the colorimetric assay described by Mosmann (1983). The insoluble formazan crystals were dissolved with isopropanol and then the absorbance was recorded at 570 nm using the microplate reader Mithras LB 940 (Berthold Technologies). The amount of formazan was directly correlated to the number of metabolically active cells. The results were expressed as a percentage of viability compared to the negative control (cells cultivated in culture medium alone), which was considered 100% viable. Data were presented as the average of three replicates (mean ± SD).

The cytotoxicity of the alloys was assessed by measuring the amount of lactate dehydrogenase (LDH) released into the culture medium when cells are damaged or under stress. The LDH assay was performed using the CytoTox96 kit (Promega, Madison, WI, USA) according to the manufacturer’s instructions. The amount of LDH released into the culture medium was recorded at 490 nm using the SPECTROstar^®^ Nano microplate reader (BMG Labtech, Ortenberg, Germany). The obtained values were directly proportional to the number of cells that have lost their cell membrane integrity and, therefore, their viability. Data were presented as an average of three replicates (mean ± SD).

### 2.5. Cell Morphology

The cell morphology was determined by optical microscopy with the help of Giemsa staining after 72 h of cell cultivation in the presence of different concentrations of the test samples. After removing the culture medium, the cells were washed with phosphate buffered saline (PBS) and fixed with cold methanol (−20 °C) for 5 min. After removing the fixative, the cells were washed with distilled water and stained with Giemsa solution for 20 min. Later, after washing the cells with distilled water, they were examined with a Zeiss Axio Observer D1 optical microscope. Giemsa staining is used to differentiate the nuclear and cytoplasmic morphology of different cell types, staining the nuclei in dark blue to purple and the cytoplasm in different shades of blue.

Cell morphology was also evaluated by fluorescence microscopy using a LIVE/DEAD Cell Viability/Cytotoxicity Kit (Molecular Probes, Invitrogen, Eugene, OR, USA) according to the manufacturer’s instructions. Briefly, after 24 h of cell incubation in standard conditions in the presence of sample extracts, cells were washed with PBS and stained with calcein-AM (2 μM) and ethidium homodimer-1 (4 μM) at room temperature for 30 min. Fluorescent images were acquired using a Zeiss Axio Observer D1 microscope and the AxioVision 4.6 software, and processed using the ImageJ 1.51 software.

### 2.6. Antibacterial Tests

#### 2.6.1. Planktonic Bacteria Model

The antimicrobial potential of the 3D-printed scaffolds was tested against both Gram-positive (*Staphylococcus aureus* (ATCC 25923)) and Gram-negative strains (*Pseudomonas aeruginosa* (ATCC 27853)). Among the most common pathogens involved in bone infections, *Staphylococcus aureus* is the leading organism along with *S. epidermidis*, *Pseudomonas aeruginosa*, *Serratia marcescens*, and *Escherichia coli* [25,26]. *S. aureus* was grown on trypticase soy agar (TSA) nutrient medium and *P. aeruginosa* on Luria Bertani agar at 37 °C. Fresh, 18 h cultures were used for obtaining standardized inoculum with a final concentration of 1 × 10^8^ colony forming units per mL (CFU/mL) in each well. Prior to seeding, UV-sterilized samples for 1 h on each side were inserted in each well. After overnight incubation, the absorbance of the supernatant was assessed at 600 nm to determine the bacterial viability using a Sunrise microplate reader (Tecan, Zürich, Switzerland).

#### 2.6.2. Bacterial Biofilm Model

The adhesion of the 2 bacterial strains was detected spectrophotometrically. Briefly, bacterial suspensions were seeded at a density of 1 × 10^8^ CFU/mL in each well in a flat-bottom 96-well plate. After 72 h, each scaffold was washed 3 times in sterile phosphate-buffered saline (PBS; pH 7.2) to remove non-adherent cells, while the bacteria attached to the samples were fixed with methanol, stained with aqueous crystal violet 1% solution, and decolorized with 33% acetic acid. The optical density of each well stained with crystal violet was measured at 495 nm using a Sunrise plate reader (Tecan).

## 3. Results and Discussion

### 3.1. Coating Morphology

**Figure 1:**
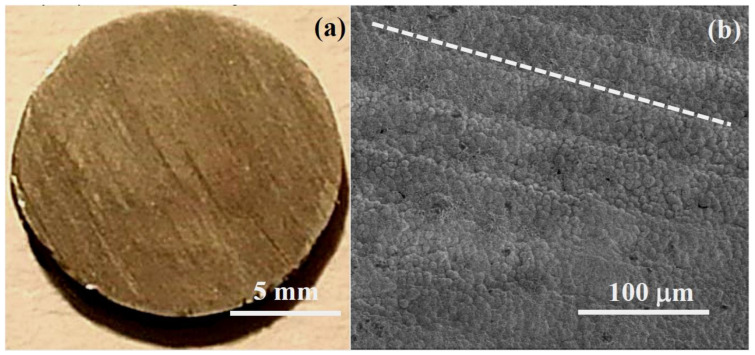
Optical (**a**) and SEM (**b**) images of Mg disk after immersion in carbonated water for 30 min, showing the formed CaCO_3_ coating.

The SEM study showed a continuous, uniform film of calcite micro-crystals with an excellent coverage of the Mg surface. Figure 1a shows an optical image of a coated disk. Immersion in carbonated water results in the homogeneous change of color of the surface, from metallic shine to a dull, matte brown surface. The parallel lines that can be observed at the disk surface are the cutting lines that preexisted in the Mg sample, indicating that the coating does not modify the surface morphology of the Mg disks at the macro-scale. Morphological observations in the micro-scale by using SEM (Figure 1b) show the formation of a textured layer of microparticles. It can be also observed the pattern of aligned cutting lines on the surface of the coated disk in the direction indicated, as a guide to the eye, by the dashed line (Figure 1b).

### 3.2. Surface Formation Mechanism

**Figure 2:**
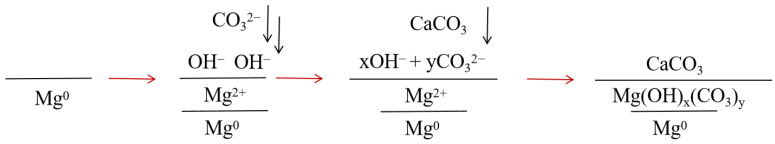
Scheme of surface reactions.

The surface chemistry of the coating is described in the scheme in Figure 2. The tentative mechanism of formation involves the substitution of hydroxyl groups at the surface of Mg by carbonate groups and the nucleation and growth of CaCO_3_. CaCO_3_ is a very important mineral compound in geological and environmental sciences. It is the most abundant biomineral, present in geological deposits and ocean sediments, and many living organisms and biominerals such as pearl or mollusk shells. Its functions include structural support (i.e., bones) and protection (i.e., shells) [27]. CaCO_3_ precipitation is not a simple process as it exists as an amorphous calcium carbonate (ACC) phase, different hydrated metastable phases, and three anhydrous crystalline polymorphs (calcite, aragonite, vaterite) [28]. Controlled mineralization of CaCO_3_ has been actively investigated [29] and further research is still needed. Most studies to date have dealt with the nucleation and crystallization of particles in solution, but comparatively few reports can be found on the formation of carbonate coatings [30]. The development of the calcium carbonate surface protection method on Mg was carried out in a preliminary phase by studying reactions in carbonated water from the native corrosion product of Mg, i.e., Mg hydroxide, brucite, and Mg(OH)_2_, to determine the formation of appropriate interfaces and of the chemical bond, by conversion from the hydroxide to the interface material of the coatings. Preliminary tests of brucite in carbonated water allowed the establishment of optimal conditions for the development of Mg hydroxycarbonates (hydromagnesite Mg_3_(CO_3_)_4_(OH)_2_·4H_2_O and giorgiosite Mg_3_(CO_3_)_4_(OH)_2_·5H_2_O) as intermediate phases for magnesium-doped calcite growth. XRD analysis and Raman microspectroscopy studies demonstrated the formation of hydromagnesite (hydroxycarbonate of magnesium) as an intermediate phase suitable for the nucleation of calcium carbonate [13]. The mechanism of formation of the coating in carbonated water containing Ca^2+^ ions is based on the dissolution of the native corrosion product of Mg, Mg(OH)_2_, the anions OH- being replaced at the oxidized metal surface (Mg^2+^) by CO_3_^2−^ anions, nucleating Mg carbonate hydrates as transient phases, with the increase of [OH-] in the aqueous solution leading to a local increase in pH, activating the precipitation of magnesian calcite. Therefore, a subsequent step in our research was to directly prove the presence of hydroxyl and carbonate groups and to determine the evolution with time of both species on the carbonated Mg surface.

### 3.3. Raman Spectroscopy

**Figure 3:**
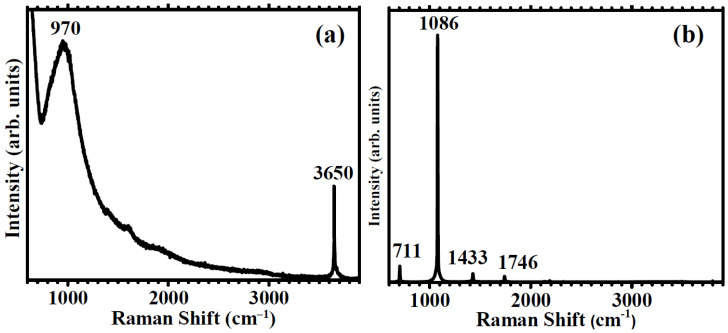
Raman spectra of (**a**) the Mg surface after 1 min immersion in carbonated water showing the presence of the hydroxyl groups stretching band at 3650 cm^−1^ and (**b**) calcite, showing the main carbonate band at 1086 cm^−1^ and secondary Raman bands of calcite.

**Figure 4:**
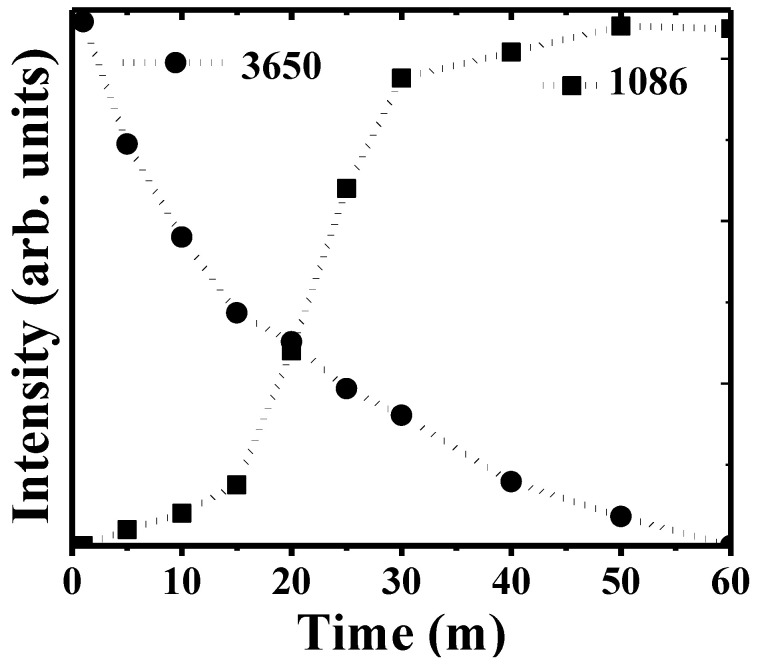
Evolution with time of immersion of Raman bands at 3650 cm^−1^ and 1086 cm^−1^ representative of the hydroxyl (OH^−^) and carbonate (CO_3_^2−^) groups.

We have used Raman spectroscopy to determine the presence of hydroxyl and carbonate groups in the coating film formed on Mg. Raman spectroscopy is a very powerful technique for the phase identification of any calcium carbonate as it can clearly identify and discriminate between ACC and each different crystalline allotrope of CaCO_3_, while also being a rapid, non-destructive technique suited for the study of thin films, suitable for mapping the spatial distribution of each carbonate phase with a lateral resolution of a few microns [31,32,33]. Moreover, Raman spectroscopy is an excellent analytic method to identify the presence of hydroxyl groups from their characteristic symmetric stretching vibration at 3600–3700 cm^−1^ [34]. Figure 3a shows the Raman spectrum of the amorphous hydroxide layer formed on Mg immediately after immersion in the carbonated water solution. The presence of the characteristic sharp band at 3650 cm^−1^ indicates the formation of a hydroxide layer at the Mg surface [34]. The Raman spectrum of calcite (Figure 3b) has a dominant band at 1086 cm^−1^ and secondary bands located at 711, 1433, and 1746 cm^−1^, assigned to the A_1g_ and E_g_ vibrational modes, respectively, of the calcite CO_3_ group [35]. We used the evolution of the intensities of the Raman bands at 3650 cm^−1^ and 1086 cm^−1^, corresponding to the presence of hydroxyl and carbonate groups, respectively, to evaluate the degree and kinetics of hydroxyl substitution with carbonate, according to the surface reaction scheme proposed in Figure 2. The evolution with the immersion time of the intensities of both Raman bands is shown in Figure 4.

Figure 4 illustrates the incorporation of carbonate groups into the coating. The intensity of the 1086 cm^−1^ vibration band grows gradually, almost linearly, immediately after immersion, until an abrupt surge is observed at 20 min immersion time. We explain this sudden increase by the homogeneous formation of crystalline calcite nuclei along the coating. Regarding the hydroxyl groups, the intensity of the 3650 cm^−1^ band decreases gradually until it practically disappears after 60 min immersion, when calcite bands remain the only Raman features.

### 3.4. Morphological Evolution (SEM, AFM) 

**Figure 5:**
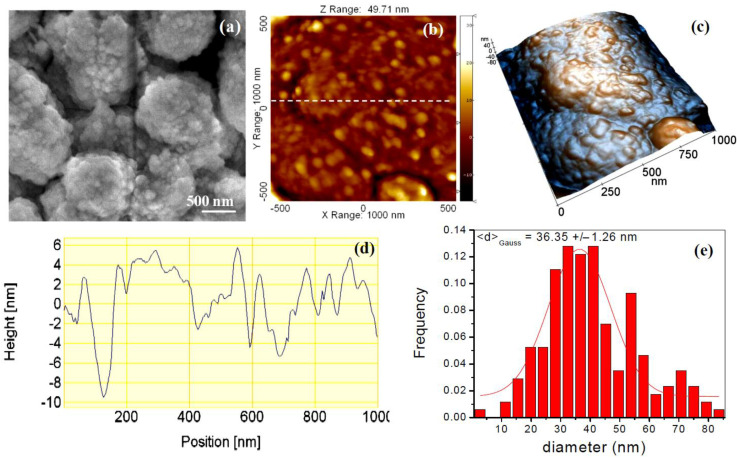
SEM (**a**); bi-dimensional (**b**) and tri-dimensional (**c**) AFM images showing the surface morphology after 20 min of immersion, scanned on a 1 μm × 1 μm region. Characteristic line scan surface profile (**d**) showing the nano-scale rugosity of the coating and histogram of the surface nanoparticles size (**e**) fitted with a Gaussian distribution.

The morphological evaluation at the micro-scale indicates how the growth of the calcite crystals layer takes place by attachment of ACC nanoparticles. Figure 5 shows the surface morphology of the carbonate coating layer formed after 20 min immersion in carbonated water. SEM images (Figure 5a) show the formation of sub-micron-sized aggregates formed by primary nanoparticles sized around 30 nm. AFM image analysis was carried out in order to determine the nano-scale topography of the coated sample immediately after immersion (Figure 5b), emphasizing the nano-scale rugosity/corrugation of the amorphous calcium carbonate (ACC) film. Figure 5 presents bi-dimensional (b, c) and tri-dimensional (d) AFM images of the Mg disk after immersion for 15 min in carbonated solution at the scale of (1 μm × 1 μm), together with an example of one characteristic line scan (surface profile) in Figure 5c. The histogram of the surface nanoparticles size is displayed in Figure 5d, fitted with a Gaussian distribution, showing a mean value of approximately 36 nm for the diameter of the protruding particles formed after 15 min of immersion in carbonated solution. The nano-scale rugosity of the ACC coating is reflected in the roughness parameters as follows: Rq = 36.9 nm and a peak-to-valley parameter, Rpv, of 182.3 nm, reflecting the nano-scale rugosity and the existence of steps between nanoparticle aggregates sized hundreds of nm. On the other hand, the AFM images demonstrate a full coverage of the Mg surface by the ACC layer.

**Figure 6:**
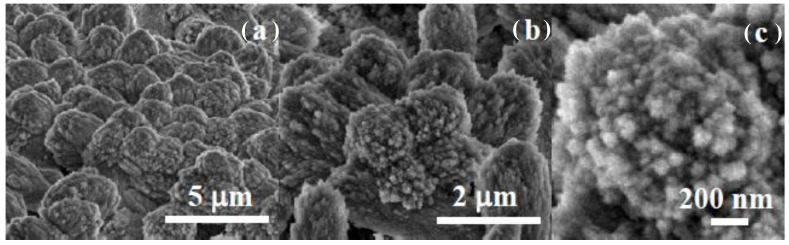
SEM images at different magnifications ((**a**), 5 µm; (**b**), 2 µm; (**c**), 200 nm) showing the nucleation of calcite nano-crystals in the carbonate layer.

The morphological evolution of the film involves the transition from the largely disordered hydroxycarbonate film to crystalline calcite [36]. The growth of calcite crystals must proceed at a homogeneous rate along the Mg surface to maintain the structural integrity of the coating, avoiding inhomogeneous or localized excessive growth of calcite crystals. The SEM images (Figure 6) taken after 20 min of immersion show the formation of calcite nanoparticles, the initial stage of crystallization of the calcite layer coating. The SEM images at different magnifications indicate that nucleation of calcite nano-crystals takes place homogeneously by the crystallization of preexisting amorphous primary nanoparticles of the coating layer. The biomimetic deposition of a crystalline microcoating on calcite using an ACC solution as a calcite precursor has been observed previously [37].

**Figure 7:**
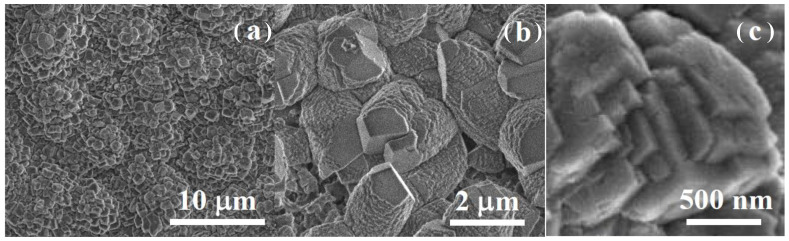
Calcite layer formed by the coalescence of growing calcite crystals: SEM ((**a**), 10 µm; (**b**), 2 µm; (**c**), 500 nm).

**Figure 8:**
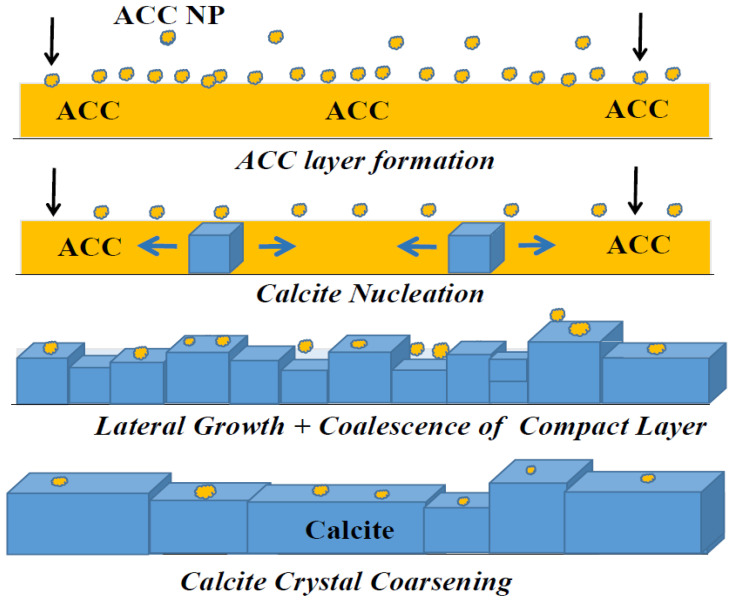
Scheme illustrating the 2D growth of calcite coating.

Figure 7 illustrates the additional growth of calcite nano-crystals after 1 h of immersion into full micro-crystals; the flat facet of one growing calcite crystal lattice is indicated by an arrow in Figure 7b. The sizes of the micro-crystals (Figure 7) correlate well with those of the secondary aggregates observed in the amorphous layer (Figure 5a). These results indicate the mechanism for the formation of a calcite micro-crystalline coating layer, which is proposed in the scheme in Figure 8. Hydroxyl substitution by carbonates and dehydration leads to an initially amorphous ACC layer fully covering the Mg surface. The nucleation of calcite nano-crystals and lateral growth within the amorphous layer leads to the coalescence of the growing calcite micro-crystals into a compact layer of intertwined calcite micro-crystals, aided by the oriented attachment of primary nanoparticles to growing calcite lattices. 

**Figure 9:**
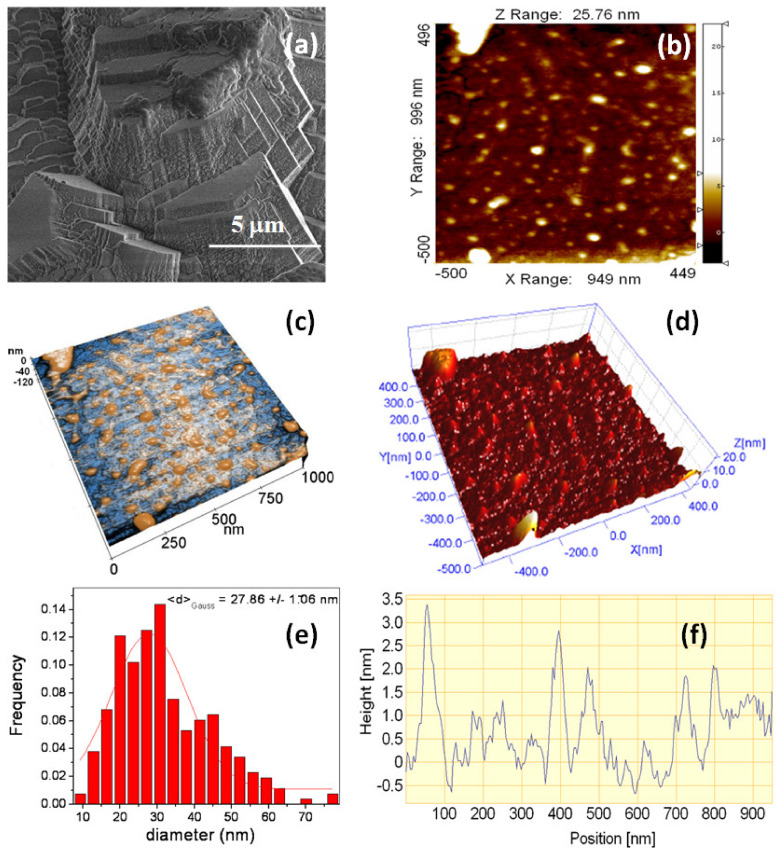
SEM (**a**); bi-dimensional (**b**) and tri-dimensional (**c**,**d**) AFM images taken on a flat region of a crystalline calcite facet; histogram of the surface nanoparticles (**e**) fitted with a Gaussian distribution; and line profile (**f**) showing the deposition of nanoparticles on the underlying calcite crystal facet.

Figure 9 presents the surface morphology of the Mg disk after the formation of a continuous coating of calcite crystals. The SEM image in Figure 9a shows the formation of euhedral crystals in the micro-scale, bi-dimensional (Figure 9b) and tri-dimensional-enhanced contrast mode and enlarged view in the z-axis (Figure 9c,d) AFM images obtained by selecting a flat area on top of one exposed calcite crystal facet, such as the one indicated by an arrow in Figure 7b, revealing a uniform structure of small particles of approximately 28 nm mean diameter, as indicated by the histogram of the surface nanoparticles fitted with a Gaussian distribution (Figure 9e). These nanoparticles appear to be the fine surface structure of the growing calcite crystals. Figure 9d,f presents the line scan profile along the horizontal dashed line in Figure 9b. The nano-scale roughness of the sample assessed in Figure 9 exhibits small roughness parameters as follows: Rq = 1.4 nm and a peak-to-valley parameter, Rpv, of 15.8 nm. 

**Figure 10:**
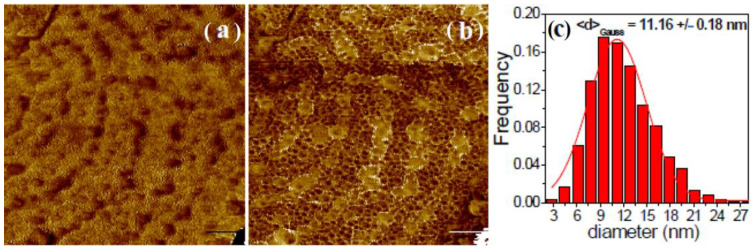
Raw bi-dimensional AFM images (unprocessed/as registered) scanned on a 1 μm × 1 μm region on a calcite crystal facet, showing the amplitude (**a**) and phase contrast (**b**) after 1 day of immersion. The small particles histogram (**c**), fitted by a Gaussian distribution, suggests a smaller mean diameter of approximately 11 nm.

Figure 10 shows the amplitude (a) and phase contrast (b) images of the same sample from Figure 9. The amplitude image (Figure 10a) reflects only the surface topography of the calcite crystals (in agreement with Figure 9). On the other hand, the phase contrast image highlights that in addition to the nanometric roughness described in Figure 9 (with the corresponding histogram in Figure 9e), calcite crystal facets are endowed with an additional sub-structure distinguishable as small black spots in Figure 10b. It can be presumed that the mosaic pattern covering the calcite crystals surface corresponds to growth by the successive attachment of amorphous calcium carbonate nanoparticles (ACC) that are incorporated into the growing calcite crystal during immersion in carbonated water. These results confirm the growth of calcite lattices by nanoparticle attachment, as illustrated in the scheme in Figure 8.

### 3.5. Cell Viability

**Figure 11:**
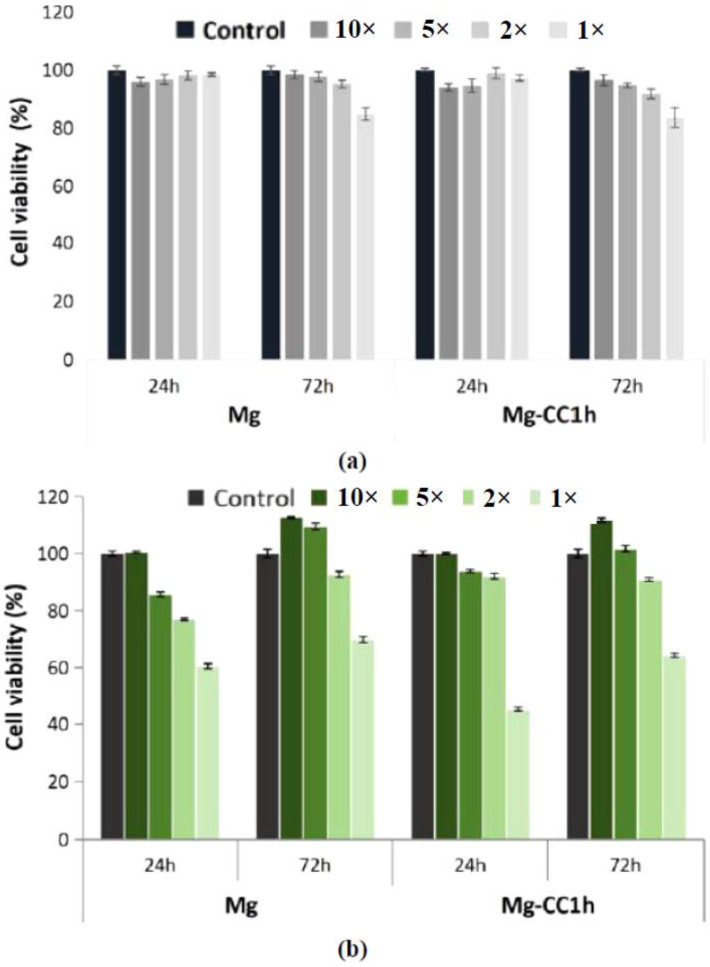
Viability of NCTC mouse fibroblasts (**a**) and SaOS-2 human osteoblasts (**b**) cultured in the presence of Mg and Mg-CC1h, for 24 h and 72 h, evaluated by the MTT test. The viability of the treated cells was obtained in reference to the negative control (untreated cells), considered to be 100% viable. The data were expressed as the average of the samples analyzed in triplicate (mean ± SD).

The cytotoxic effect was evaluated on two cell lines (NCTC and SaOS-2) by the MTT test, which evaluates the activity of mitochondrial dehydrogenases, and by the LDH test, which investigates the integrity of the cell membrane by quantifying the amount of the LDH enzyme released into the culture medium following cell lysis. The results of the MTT test on NCTC mouse fibroblasts showed that both materials, Mg and Mg coated with calcite-type calcium carbonate after 1 h of immersion, named Mg-CC1h, did not induce a cytotoxic effect. The cell viability percentages were higher than 80% (non-cytotoxic effect) for the tested dilutions (1×, 2×, 5×, and 10×) for Mg and Mg-CC1h at both exposure times (24 and 72 h) (Figure 11a). Different results were obtained in the case of SaOS-2 human osteoblasts (Figure 11b). On this cell type, only the 2×, 5×, and 10× dilutions were cytocompatible at both exposure times, with values of cell viability percentages greater than 90% (Figure 11b). The uncovered sample induced slightly lower cell viability values for 5× and 2× dilutions at 24 h (77.16% and 85.80%, respectively). For the 1× dilution, both extracts induced moderate cytotoxicity, with cell viability percentages lower than 70% (Figure 11b).

**Figure 12:**
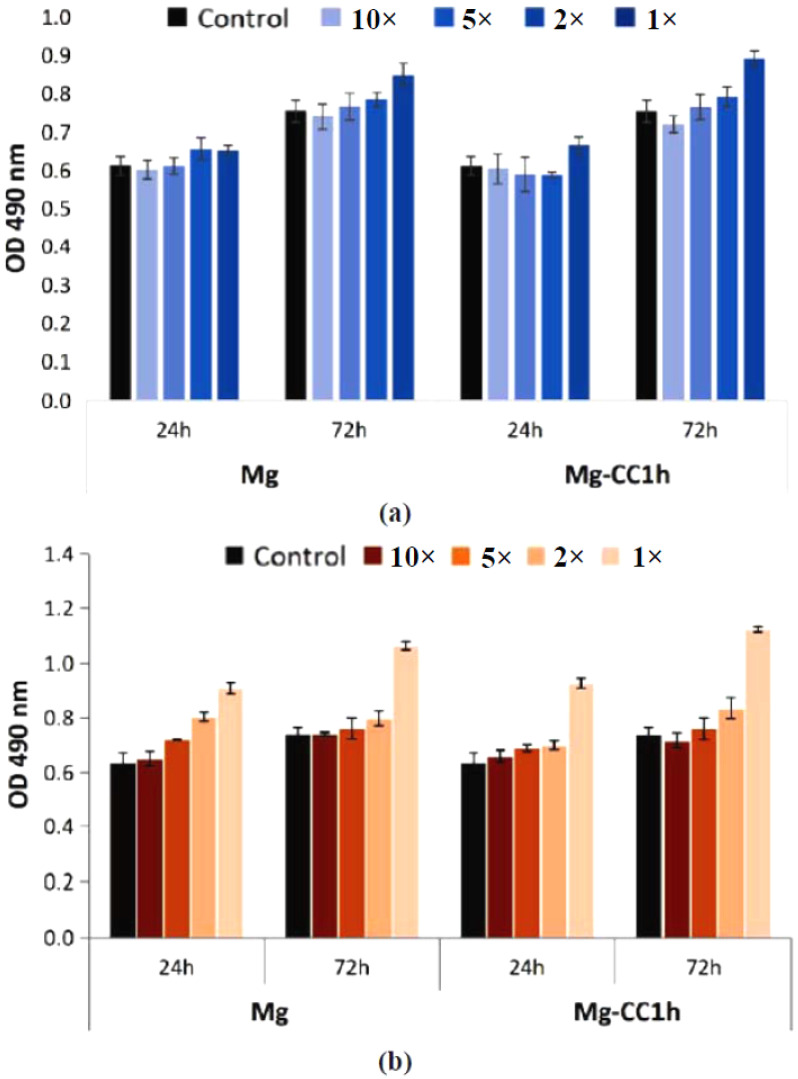
The levels of LDH released into the culture medium by NCTC fibroblasts (**a**) and SaOS-2 human osteoblasts (**b**) cultured in the presence of Mg and Mg-CC1h for 24 h and 72 h. The data were expressed as the average of the samples analyzed in triplicate (mean ± SD).

Cells cultured in the presence of Mg and Mg-CC1h were also investigated from the point of view of cell membrane integrity by measuring the amount of LDH released in the culture medium. The obtained results indicated a different cytotoxic effect depending on the cell type used. Thus, NCTC mouse fibroblasts cultured in the presence of both materials showed low levels of LDH released in the culture medium, similar to those of the control sample, at both exposure times for the tested dilutions (2×, 5×, and 10×), indicating that the integrity of the cell membrane was not affected and thus neither was cell viability (Figure 12a). In the case of SaOS-2 human osteoblasts, the level of LDH released was reduced and comparable to that measured in the case of the control sample only for the 2×, 5×, and 10× dilutions, suggesting that the two samples (Mg and Mg-CC1h) are not cytotoxic (Figure 12b). However, increased levels of LDH were observed in the culture medium for both samples at both test times for the 1× dilutions (Figure 12b). These results were correlated with the results obtained from the MTT test, indicating a cytotoxic effect of both samples only at the 1× dilution in the case of tests performed on human SaOS-2 osteoblasts.

### 3.6. Cell Morphology

**Figure 13:**
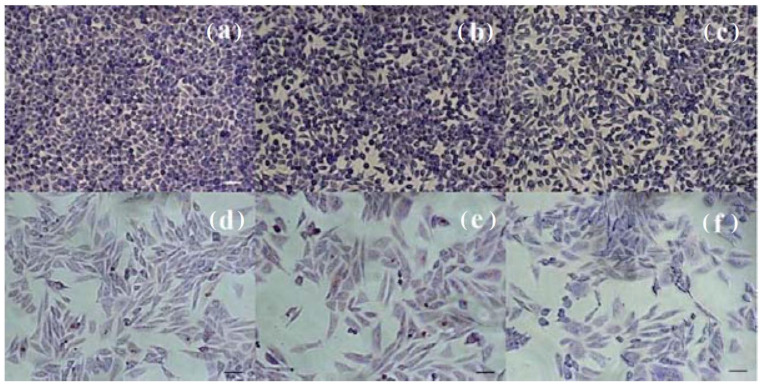
Optical microscopy images of NCTC mouse fibroblasts: untreated (**a**) and treated with dilutions of the Mg (**b**) and Mg-CC1h samples (**c**) for 72 h, as well as SaOS-2 osteoblasts: untreated (**d**) and treated with the dilutions of the Mg (**e**) and Mg-CC1h samples (**f**) for 72 h. The untreated cell culture was used as a control (Giemsa staining). Bar = 50 μm.

The cells showed normal appearance and density, similar to those of the control sample. In the case of NCTC mouse fibroblasts treated with extracts of Mg and Mg-CC1h, no significant changes in morphology and cell density were observed for 1× dilutions (Figure 13b,c), which were similar to those of the control sample (Figure 13a), with the culture almost reaching the cell monolayer. The cells presented a normal, slightly polygonal appearance, with 2–3 cytoplasmic extensions and fine cytoplasm. The results obtained on SaOS-2 human osteoblasts were similar to those observed on NCTC fibroblasts. The cells presented a normal, osteoblast-like appearance with a single nucleus and numerous nucleoli (Figure 13e,f). Also, the cell density was similar to that of the control sample (Figure 13d), with the culture covering around 70–75% of the surface of the well.

**Figure 14:**
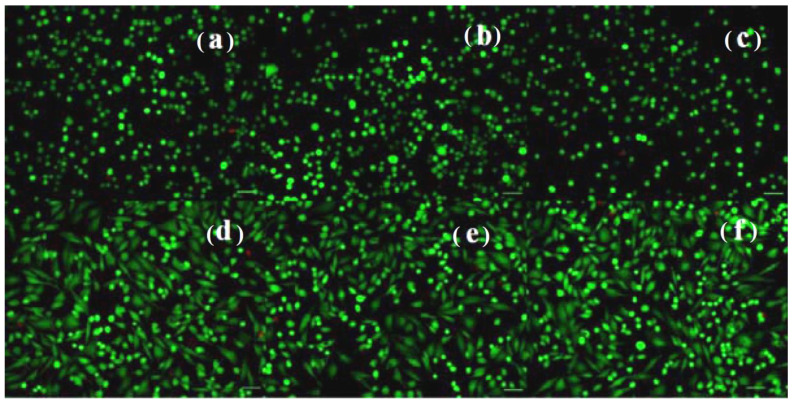
Fluorescent staining with calcein-AM (green) and ethidium homodimer-1 (red) of NCTC live and dead cells, untreated (**a**) and treated with solutions of the Mg (**b**) and Mg-CC1h samples (**c**) for 24 h; and SaOS-2 osteoblasts untreated (**d**) and treated with dilutions of the Mg (**e**) and Mg-CC1h samples (**f**) for 24 h. Scale bar = 50 μm.

The cell morphology and viability were also assessed by fluorescence microscopy following the staining of live NCTC and SaOS-2 cells with calcein (green) and of dead cells with ethidium homodimer (red) after 24 h of treatment with the Mg and Mg-CC1h samples. NCTC murine fibroblasts maintained their viability after 24 h of treatment and few dead cells were observed (Figure 14b,c). In addition, the treated cells showed no morphological changes, maintained their normal morphology, and showed a density similar to that of untreated cells (Figure 14a). Comparable results, with the preservation of the normal phenotype and cell density similar to that of the control, were observed for the SaOS-2 cells treated with Mg- and carbonate-coated samples (Figure 14d–f). These results were in line with those obtained by the quantitative MTT and LDH assays.

### 3.7. Antimicrobial Effect

Antimicrobial potential evaluation against both Gram-positive (*Staphylococcus aureus* (ATCC 25923)) and Gram-negative (*Pseudomonas aeruginosa* (ATCC 27853)) strains demonstrated that the coated samples significantly inhibited bacterial adhesion and biofilm formation compared to the untreated control.

#### 3.7.1. Planktonic Growth

**Figure 15:**
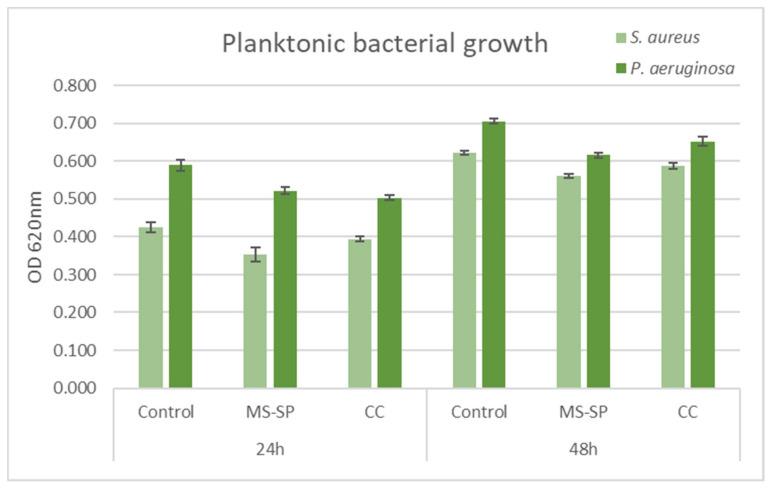
Microbial planktonic growth in the presence of Mg and Mg-CC1h in time (24 and 48 h). Experimental data are reported as mean ± SD. *n* = 3.

The planktonic bacterial growth was monitored in time by means of spectrophotometric measurements. After 24 h of cultivation on the 3D-printed scaffolds, *S. aureus* registered a decrease when compared to the untreated control, MS-SP being more efficient in inhibiting bacterial viability. The trend was maintained also at 48 h, with *S. aureus* being more susceptible when compared to *P. aeruginosa* (Figure 15). Nevertheless, both types of scaffolds interfered with the planktonic microbial growth when compared to the strain control cultivated in a broth medium.

#### 3.7.2. Anti-Adherent Potential of 3D Scaffolds

**Figure 16:**
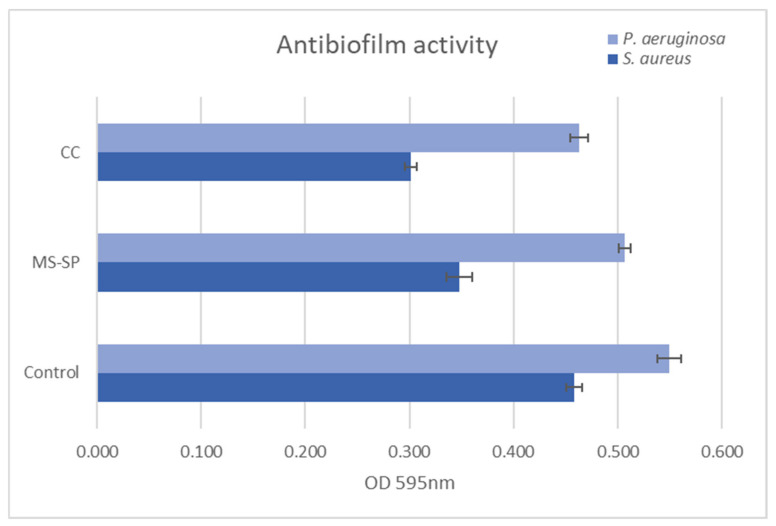
*S. aureus* and *P. aeruginosa* biofilm development onto Mg and Mg-CC1h at 72 h. Experimental data are reported as mean ± SD. *n* = 3.

In terms of inhibiting microbial adhesion, the 3D-printed scaffolds showed great potential after 72 h of co-cultivation. The results indicate that the Mg-based scaffolds significantly inhibited bacterial adhesion and biofilm formation compared to the untreated control. The OD values of the CC scaffold were significantly lower for both strains tested (Figure 16). Among the two microorganisms, *S. aureus* proved to be more susceptible, probably due to intrinsic bacterial architecture, the lack of an outer membrane, and LPS. It is known that Gram-positive bacteria are more susceptible to vegetal compounds when compared to Gram-negative bacteria [38]. The increase in pH caused by Mg^2+^ concentration and osmolality as a major factor contributing to antibacterial and antibiofilm activity has been reported [39].

## 4. Conclusions

In conclusion, the immersion of biodegradable Mg in carbonated water solution at room temperature leads to the formation of a protective biomimetic calcite coating by a simple surface mineralization method. The CaCO_3_ mineralization mechanism involves the substitution of hydroxyl groups at the surface of Mg by carbonate groups, the formation of an amorphous ACC film, and the nucleation and growth of calcite crystals. SEM images showed a continuous, uniform film of calcite micro-crystals with excellent Mg surface coverage. The morphology, cytocompatibility, and cell viability of the calcite-coated Mg samples were evaluated in order to apply calcium carbonate as a biomaterial coating on Mg implants. Preliminary results show that the simple green synthesis method used leads to obtaining coated Mg samples with desired properties of cell compatibility and antibacterial protection. Calcite coatings generated by a single-step process could be used on resorbable Mg implants with the possibility of mitigating the rate of degradation during the healing period.

## Data Availability

The data presented in this study are available on request from the corresponding author.
